# Fatal Hemorrhagic Rupture of a Cystic Hepatic Hemangioma in a 27-Week Preterm Neonate

**DOI:** 10.7759/cureus.105144

**Published:** 2026-03-13

**Authors:** Omayma El Athmani, Salah Saghir, Mustapha Azzakhmam, Anas Ayad, Rachid Abilkassem

**Affiliations:** 1 Department of Pediatrics, Mohammed V Military Hospital, Rabat, MAR; 2 Department of Anatomic and Cytopathology, Mohammed V Military Hospital, Rabat, MAR

**Keywords:** cystic hepatic hemangioma, extreme prematurity, hepatic vascular tumor, neonatal hemorrhage, preterm neonate, ruptured hemangioma

## Abstract

Hepatic hemangiomas are uncommon vascular tumors in the neonatal period and are rarely reported in extremely preterm infants. Cystic variants are exceptionally rare and may exhibit atypical imaging features, making early diagnosis challenging. Spontaneous rupture with massive hemorrhage represents a life-threatening complication. We report the case of a male neonate born at 27 weeks of gestation with a birth weight of 900 g who was initially admitted to the neonatal intensive care unit for respiratory distress syndrome. During hospitalization, the infant developed recurrent severe anemia associated with progressive hepatomegaly and abdominal distension requiring multiple packed red blood cell transfusions. Clinical examination revealed marked abdominal enlargement. Abdominal radiography and contrast-enhanced computed tomography demonstrated a large multiloculated cystic hepatic mass. The clinical course rapidly deteriorated with acute hemorrhagic anemia and progressive abdominal enlargement, ultimately resulting in death. Histopathological examination of a postmortem liver biopsy confirmed a ruptured cystic hepatic hemangioma. In very preterm neonates, unexplained anemia associated with hepatomegaly should prompt consideration of rare hepatic vascular tumors. Although exceedingly rare, ruptured cystic hepatic hemangioma carries a poor prognosis in this vulnerable population.

## Introduction

Neonatal liver tumors are rare, with hepatic hemangiomas representing the most frequently reported benign vascular hepatic lesions in infancy [1,2]. These tumors are typically identified in term neonates or during early infancy and usually follow a favorable clinical course [3]. However, their clinical presentation may vary widely depending on tumor size, number, and associated complications.

Cystic hepatic hemangiomas are an unusual morphological variant that may delay diagnosis because of their atypical radiologic appearance [2]. Although most hepatic hemangiomas remain uncomplicated, spontaneous rupture is an exceptional but catastrophic event that may result in massive intra-abdominal hemorrhage and hemodynamic collapse [4-6].

Infantile hepatic hemangiomas belong to the spectrum of vascular tumors encountered in infancy and may present with variable clinical manifestations depending on their size and associated complications [7,8].

In extremely preterm neonates, diagnostic challenges are amplified by nonspecific clinical manifestations and overlapping neonatal morbidities. Data regarding ruptured cystic hepatic hemangioma in this population remain extremely limited. We report a fatal case in an extremely preterm infant, highlighting the diagnostic difficulties and severe clinical course associated with this rare entity.

## Case presentation

A male neonate was delivered at 27 weeks of gestation by spontaneous vaginal delivery to a 31-year-old mother (gravida 3, para 2). Pregnancy was complicated by unexplained preterm labor in a context suggestive of maternal infection. No hepatic lesion was detected on routine prenatal ultrasonography.

At birth, the infant weighed 900 g with Apgar scores of 9, 10, and 10 at 1, 5, and 10 minutes, respectively. The neonate was admitted to the neonatal intensive care unit for management of respiratory distress syndrome related to prematurity.

During hospitalization, the infant progressively developed abdominal distension associated with recurrent episodes of severe anemia without evidence of external bleeding. The total duration of hospitalization was 18 days.

**Figure 1:**
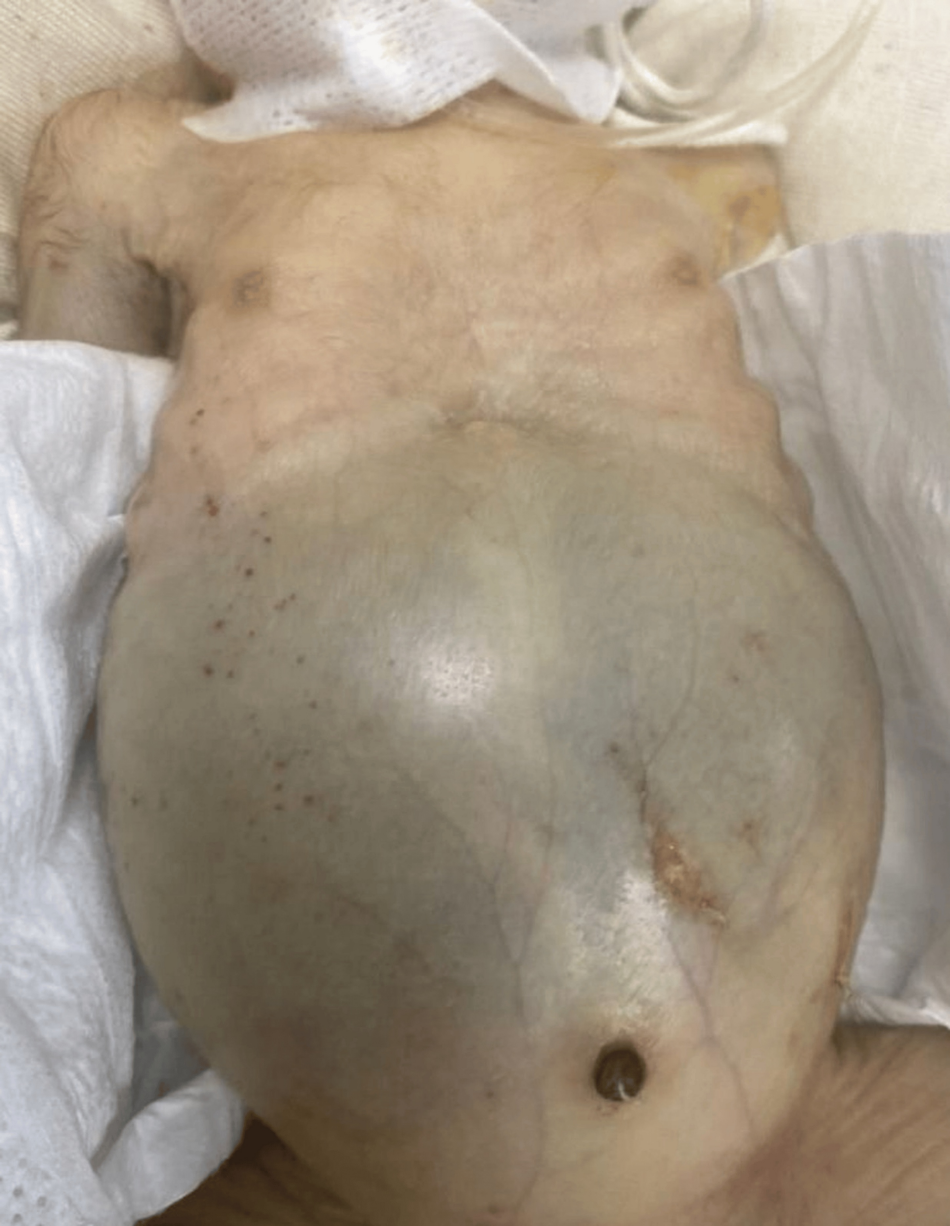
Severe abdominal distension in a very preterm neonate Clinical photograph of an extremely preterm neonate demonstrating massive abdominal distension with a tense abdominal wall. Superficial abdominal venous collateral circulation is visible, suggestive of portal hypertension secondary to severe hepatomegaly.

Clinical examination revealed massive abdominal enlargement with a tense abdominal wall and visible superficial abdominal venous collateral circulation suggestive of portal hypertension (Figure 1). 

Laboratory investigations demonstrated severe normocytic anemia with hemoglobin levels dropping to 5.1 g/dL, requiring seven packed red blood cell transfusions. Platelet counts remained within the normal neonatal range, and inflammatory markers were not significantly elevated. Liver enzymes were moderately elevated. The main laboratory findings during hospitalization are summarized in Table 1.

**Table 1:** Laboratory findings during hospitalization Values are presented using neonatal reference ranges. ALT, alanine aminotransferase; AST, aspartate aminotransferase.

Parameter	Result	Reference range
Hemoglobin (g/dL)	5.1	14-22
Hematocrit (%)	15	42-65
Red blood cells (×10¹²/L)	1.8	4.0-6.0
White blood cells (×10⁹/L)	14.2	9-30
Neutrophils (%)	56	40-70
Platelet count (×10⁹/L)	168	150-400
C-reactive protein (mg/L)	3.5	<10
Prothrombin time (%)	64	70-100
AST (U/L)	105	<40
ALT (U/L)	82	<40
Total bilirubin (mg/dL)	2.6	<1.2
Direct bilirubin (mg/dL)	0.8	<0.3

Abdominal ultrasonography was performed as the initial imaging modality and demonstrated hepatomegaly with a heterogeneous hepatic lesion. However, precise characterization of the lesion was limited due to the large size of the mass and poor acoustic window in this extremely preterm neonate.

**Figure 2:**
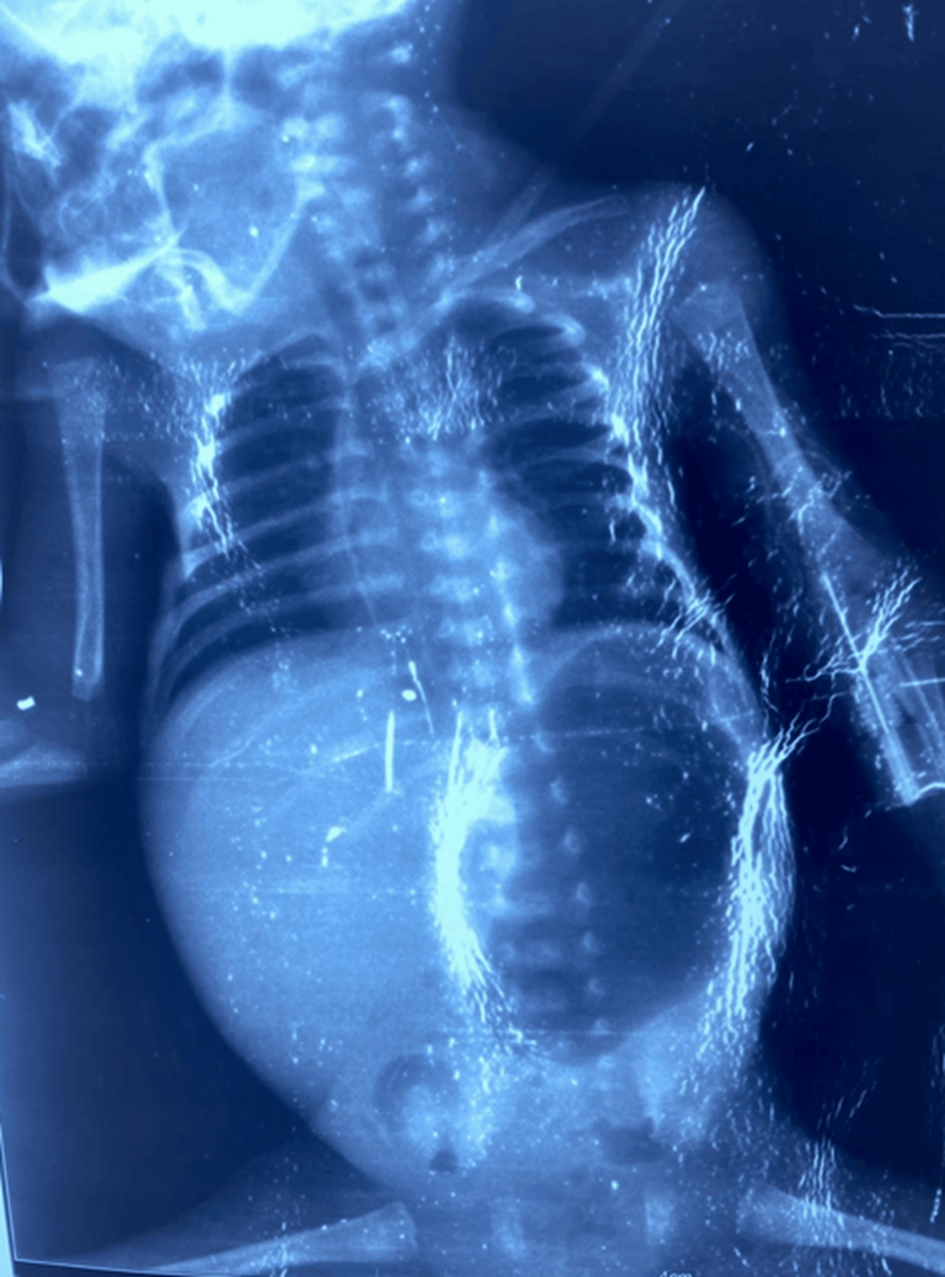
Chest and abdominal radiograph showing massive abdominal enlargement Chest and abdominal radiograph demonstrating marked abdominal enlargement with significant mass effect and compression of the right hemithorax caused by severe hepatomegaly.

Chest and abdominal radiography demonstrated marked abdominal enlargement with mass effect and compression of the right hemithorax consistent with severe hepatomegaly (Figure 2).

**Figure 3:**
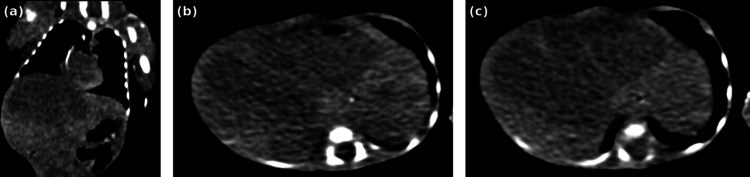
Contrast-enhanced CT demonstrating a multiloculated cystic hepatic mass with hemorrhagic features Contrast-enhanced CT images of the abdomen demonstrating a large multiloculated cystic hepatic lesion that occupies a significant portion of the liver parenchyma. (a) Coronal CT reconstruction showing a large cystic hepatic mass with multiple loculations. (b) Axial CT section demonstrating heterogeneous density within the lesion. (c) Axial CT image showing hyperdense areas suggestive of intralesional hemorrhage. CT, computed tomography.

Further evaluation with contrast-enhanced computed tomography revealed a large multiloculated cystic hepatic lesion occupying a significant portion of the liver parenchyma with internal septations and heterogeneous density. Hyperdense areas were suggestive of intralesional hemorrhage (Figures 3a-3c). 

Despite intensive supportive management, the infant developed rapid enlargement of the hepatic mass, severe anemia, and hemodynamic instability. The rapid clinical deterioration precluded consideration of medical or interventional therapies, and the clinical condition progressed to death.

**Figure 4:**
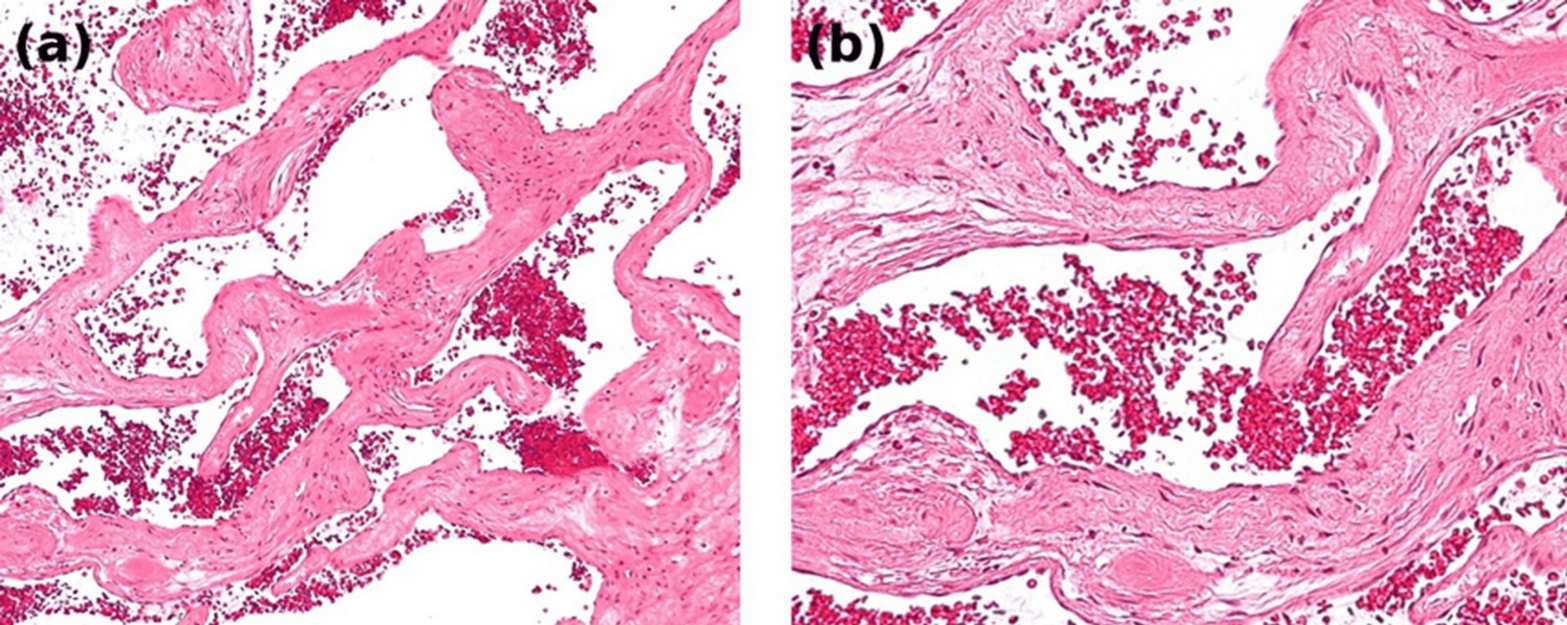
Histopathological findings of hepatic hemangioma with intralesional hemorrhage Histopathological examination of the hepatic lesion (hematoxylin and eosin stain) (a) Low-power view showing dilated vascular channels filled with erythrocytes consistent with hepatic hemangioma. (b) High-power view demonstrating vascular spaces lined by flattened endothelial cells associated with intralesional hemorrhage.

A postmortem liver biopsy was obtained. Histopathological examination demonstrated dilated vascular channels lined by flattened endothelial cells consistent with hepatic hemangioma. Intralesional hemorrhage was observed, supporting the diagnosis of hemorrhagic rupture in the appropriate clinical context (Figures 4a, 4b).

## Discussion

Hepatic hemangiomas are the most common benign vascular liver tumors in infancy but remain uncommon in extremely preterm neonates [3,4]. Cystic variants represent an unusual morphological presentation and may pose diagnostic challenges because of their atypical imaging features.

Spontaneous rupture of hepatic hemangiomas is a rare but potentially fatal complication that can lead to massive intra-abdominal hemorrhage and rapid hemodynamic deterioration [5,6]. The mechanisms underlying rupture are not completely understood but may involve fragile vascular architecture combined with rapid tumor expansion.

In extremely preterm neonates, clinical manifestations such as anemia and abdominal distension are nonspecific and often attributed to more common neonatal conditions such as intraventricular hemorrhage, necrotizing enterocolitis, or sepsis. This overlap may delay recognition of rare hepatic vascular tumors.

Imaging plays a crucial role in the diagnostic evaluation. Ultrasonography is generally the first-line modality in neonates, whereas cross-sectional imaging such as computed tomography or magnetic resonance imaging allows better characterization of hepatic lesions and their complications [7-9].

In the present case, contrast-enhanced CT imaging demonstrated a large multiloculated cystic hepatic lesion occupying a significant portion of the liver parenchyma with internal septations and heterogeneous density. Hyperdense areas within the lesion were suggestive of intralesional hemorrhage.

Histopathological examination revealed dilated vascular channels lined by flattened endothelial cells consistent with hepatic hemangioma. Extensive intralesional hemorrhage was observed within the lesion. In the clinical context of progressive abdominal distension and severe anemia, these findings supported the diagnosis of hemorrhagic rupture.

Reported therapeutic strategies for hepatic hemangiomas include medical therapy such as corticosteroids or beta-blockers, transcatheter embolization, and surgical resection [10]. However, in extremely low-birth-weight infants, these interventions may be limited by clinical instability and technical constraints.

To our knowledge, reports of ruptured hepatic hemangioma in extremely preterm neonates remain exceptionally rare. This case expands the clinical spectrum of neonatal hepatic hemangiomas by illustrating an unusual cystic presentation complicated by hemorrhagic rupture in an extremely low-birth-weight infant.

## Conclusions

Ruptured cystic hepatic hemangioma is an exceptionally rare but catastrophic condition in extremely preterm neonates. The diagnosis may be challenging because clinical manifestations such as anemia and abdominal distension are nonspecific and may mimic more common neonatal conditions.

This case highlights the importance of considering hepatic vascular tumors in very preterm infants presenting with unexplained anemia and progressive hepatomegaly. Early imaging evaluation, particularly ultrasonography followed by cross-sectional imaging when necessary, may help establish the diagnosis.
